# The relationship between family capital and academic burnout among undergraduate sports majors: the mediating role of academic self-efficacy

**DOI:** 10.3389/fpsyg.2026.1989620

**Published:** 2026-10-29

**Authors:** Yang Lan, Jun Tong, Binyang Li, Shengtao Wang, Kui Dong

**Affiliations:** 1 Kunming Medical University, Kunming, China; 2 Kunming City College, Kunming, China

**Keywords:** academic burnout, academic self-efficacy, family capital, higher education, mediation, undergraduate sports majors

## Abstract

**Background:**

Academic burnout is a concern among undergraduate sports majors. Although previous studies have emphasized training- and school-related stressors, less is known about the role of family resources and related psychological mechanisms.

**Objective:**

This study examined the associations among family capital, academic self-efficacy, and academic burnout, including differences across family capital dimensions and the mediating role of academic self-efficacy.

**Methods:**

This cross-sectional study included 325 first- and second-year students enrolled in sports-related degree programs at a sports university in Yunnan Province, China. Pearson correlation, one-way ANOVA, and mediation analysis using PROCESS Model 4 with 5,000 bootstrap resamples were conducted.

**Results:**

Family capital was positively associated with academic self-efficacy (r = 0.554, *p* < 0.001) and negatively associated with academic burnout (r = −0.575, *p* < 0.001). Cultural and social capital were significantly associated with higher self-efficacy and lower burnout, whereas economic capital was not significantly associated with burnout. Students with lower family capital reported lower self-efficacy and higher burnout. Academic self-efficacy partially mediated the association between family capital and burnout (B = −0.52, 95% CI [−0.66, −0.40]), accounting for 44.33% of the total effect.

**Conclusion:**

Family capital was associated with academic burnout among undergraduate sports majors, with stronger associations for cultural and social capital than for economic capital. Academic self-efficacy represented a partial mediating pathway. Universities should provide targeted academic guidance, learning support, and educational and career information for students with limited family resources and lower academic self-efficacy.

## Introduction

University students’ learning adaptation and psychological development are increasingly recognized as important concerns in higher education research. Academic burnout refers to a negative psychological state characterized by low mood, academic withdrawal, and reduced academic accomplishment in response to sustained academic pressure. It typically manifests as declining learning interest, reduced classroom participation, academic procrastination, self-denial, and helplessness regarding academic outcomes (Maslach et al., 2001; Schaufeli et al., 2002; Lian et al., 2005). A cross-sectional survey of 22,983 Chinese university students reported that approximately 59.9% experienced varying degrees of academic burnout, with nearly 5% reporting severe to extremely severe symptoms (Liu et al., 2023). Latent profile research further indicates substantial heterogeneity in academic burnout among Chinese university students, with low-, moderate-, and high-burnout profiles accounting for 18.15%, 50.88%, and 30.97%, respectively (Li et al., 2026). This issue has important academic consequences. A meta-analysis by Madigan and Curran (2021) showed that student burnout was significantly and negatively associated with academic achievement, and that emotional exhaustion, academic disengagement, and reduced efficacy were all linked to poorer academic performance. Recent studies suggest that academic burnout emerges from the combined influence of academic stress, motivational depletion, reduced learning engagement, and weakened competence beliefs (Ma, 2024; Qin et al., 2025). For example, Wang et al. (2024) found that academic self-efficacy negatively predicted academic burnout among university students, with intrinsic motivation and learning engagement serving as sequential mediators. Cong et al. (2024) also identified learning engagement and academic self-efficacy as important psychological and behavioral factors associated with students’ academic burnout. Thus, students’ vulnerability to burnout depends not only on external academic pressure but also on their competence beliefs, learning engagement, and psychological resources.

Academic burnout among undergraduate sports majors is particularly complex and context-dependent. Their learning activities span classrooms, training fields, and competitive settings, where coursework, specialized training, skill assessment, physical exertion, competition performance, and injury risk jointly shape their daily stressors. Previous studies have shown that student-athletes must continuously balance academic commitments with athletic training demands, which may expose them to time conflicts, physical fatigue, role pressure, and psychological adjustment difficulties (Dorsch et al., 2016; Thompson et al., 2024; De Maio et al., 2025). Exhaustion experienced in the athletic domain may also spill over into the academic domain. Xu et al. (2025) found that athletic burnout among university student-athletes significantly predicted academic burnout, with emotion regulation and sleep quality serving as mediators. However, sports majors exposed to similar academic and training pressures may still differ in their levels of academic burnout. Recent studies have shown that social and psychological resources help explain such differences: parental, teacher, and peer support are associated with behavioral engagement and school burnout among student-athletes; life stress may influence athletic burnout through mindfulness and cognitive emotion regulation; and success expectations and parental emotional support may affect the developmental trajectories of athletic burnout and school burnout (Kuokkanen et al., 2024; Ma et al., 2025; Saarinen et al., 2025).

Family capital provides a useful lens for explaining variation in academic burnout among undergraduate sports majors. Drawing on Bourdieu’s theory of capital, field, and habitus, family economic, cultural, and social capital can be converted within the educational field and shape students’ perceptions of learning, competition, failure, and future development (Bourdieu, 2011; Bourdieu and Passeron, 1990). Conceptually, family capital differs from family functioning and emotional support. Family functioning reflects family relationships and organization (Rhodes et al., 2024), whereas emotional support refers to affective encouragement and understanding (Bueno et al., 2022). Family capital, by contrast, concerns the economic, cultural, and social resources available within the family. Family background may influence students’ learning engagement, academic performance, professional identity, and psychological adjustment through economic security, cultural climate, educational expectations, social networks, and emotional support (Qiu and Ye, 2023; Djekourmane et al., 2025; Li et al., 2024). For undergraduate sports majors, family capital may include economic support, exposure to family sports culture, occupational resources, social networks, and access to information relevant to professional development (Wheeler, 2012; Dorsch et al., 2016). Failure in sports-related learning is often public and embodied; unsuccessful skill demonstrations, poor physical fitness test results, and competition losses may make competence differences highly visible. Family capital may therefore influence how students make sense of such failure experiences. Existing studies indicate that parenting styles may shape students’ learning behaviors through causal attribution and self-efficacy; students who attribute failure to low ability are more likely to experience helplessness, whereas positive family functioning and supportive parent–child relationships may reduce academic burnout by enhancing academic self-efficacy (Li et al., 2023; Sun et al., 2025). The capital perspective therefore complements research on family functioning and emotional support by shifting attention from relational quality and affective exchanges to the economic, cultural, and social resources that families possess and can mobilize to support students’ educational adaptation.

Based on these considerations, this study aimed to examine the associations among family capital, academic self-efficacy, and academic burnout among undergraduate sports majors. Specifically, we examined whether the associations with academic burnout differed across the economic, cultural, and social dimensions of family capital and whether academic self-efficacy mediated the association between overall family capital and academic burnout. Accordingly, the following hypothesis was proposed:

*H1*: Family capital is negatively associated with academic burnout among undergraduate sports majors.

Academic self-efficacy may serve as a key psychological mechanism linking family capital to academic burnout. It refers to students’ judgments of their capacity to complete coursework, master professional skills, regulate learning strategies, and cope with academic setbacks (Artino, 2012; Zajacova et al., 2005). Self-efficacy theory suggests that individuals’ beliefs about their abilities influence their effort, persistence, and behavioral choices when they encounter difficulties (Bandura, 1997). Higher academic self-efficacy may help students cope with challenges and sustain learning engagement, whereas lower academic self-efficacy may weaken learning motivation and increase procrastination, avoidance, emotional exhaustion, and reduced accomplishment (Wang et al., 2024; Rad et al., 2025). Previous studies on family-related factors have shown that family functioning, family cultural capital, social support, and parental involvement are associated with higher academic self-efficacy, and that stronger academic self-efficacy is linked to lower academic burnout, greater learning motivation, stronger learning engagement, and better academic adjustment (Brouwer et al., 2016; Wang et al., 2024; Chen et al., 2025; Yang et al., 2025; Zhu, 2025). For example, Yang et al. (2025) found that academic self-efficacy mediated the relationship between family functioning and academic burnout. Although family functioning is conceptually distinct from family capital, this evidence suggests that family-related conditions may be associated with academic burnout partly through self-efficacy. For undergraduate sports majors, greater family capital may provide access to problem-solving resources and a clearer sense of certainty about future development. This sense of certainty may strengthen students’ perceived control over academic tasks and training difficulties, thereby enhancing their academic self-efficacy. Among sports majors, competence beliefs may be shaped by course grades, training performance, skill assessments, physical fitness levels, and competition results. Academic self-efficacy may therefore be central to explaining variation in academic burnout within this group. Accordingly, this study proposes the following hypotheses:

*H2*: Family capital positively predicts academic self-efficacy among undergraduate sports majors.

*H3*: Academic self-efficacy negatively predicts academic burnout among undergraduate sports majors.

The influence of family capital on academic burnout may operate through students’ psychological processes. Economic capital may support learning, training, and competition by providing access to training equipment, competition expenses, and extracurricular training resources. Cultural capital may shape students’ understanding of professional value, frustration, and developmental pathways through the family educational climate, parents’ educational experiences, and exposure to sports culture. Social capital may help students obtain information about further education, employment, and career development through relatives’ occupational resources, social networks, and industry information channels (Wheeler, 2012; Sheridan et al., 2014; Djekourmane et al., 2025). Whether these resources reduce academic burnout may depend on their transformation into competence beliefs when students face academic and training difficulties. Students with higher academic self-efficacy are more likely to interpret course pressure, training setbacks, and skill failures as manageable challenges. By contrast, students with limited family capital and weaker academic self-efficacy may be more vulnerable to low mood, academic withdrawal, and reduced accomplishment (Wang et al., 2024; Rad et al., 2025). Existing studies have examined the associations among family resources, academic self-efficacy, and academic burnout (Wang et al., 2024; Öztürk Çelik, 2025; Yang et al., 2025; Sun et al., 2025). However, the pathway through which family capital influences academic burnout via academic self-efficacy among undergraduate sports majors remains insufficiently understood. Therefore, this study proposes the following hypothesis:

*H4*: Academic self-efficacy mediates the relationship between family capital and academic burnout among undergraduate sports majors.

Existing research has mainly examined burnout among sports majors from the perspectives of training stress, athletic burnout, and school support, but has paid less attention to how family resources shape students’ psychological processes related to learning. By integrating family capital, academic self-efficacy, and academic burnout into a unified model, the present study extends this literature from a predominantly training- and school-related perspective to a family-resource perspective. The findings may provide evidence to help universities identify students with limited family capital, strengthen their academic self-efficacy, and reduce their risk of academic burnout.

## Methods

### Research design

This study used a cross-sectional survey design. An anonymous online questionnaire was used to examine the association between family capital and academic burnout among undergraduate sports majors in China and to test whether academic self-efficacy mediated this association. The questionnaire collected demographic information, family capital indicators, and scale-based measures of academic self-efficacy and academic burnout. The study was reported in accordance with the STROBE guidelines (von Elm et al., 2007).

### Participants

Participants were first- and second-year undergraduate students majoring in sports-related disciplines at a sports university in Yunnan Province, China. First- and second-year students were selected to reduce heterogeneity associated with upper-year internships, career preparation, and graduation-related academic demands, which may independently affect academic burnout. A stratified cluster random sampling procedure was used. Students were first stratified by grade and sport specialization. Within each stratum, eligible classes were assigned identification numbers, and classes were randomly selected using a computer-generated random-number procedure. All eligible students in the selected classes were invited to participate. The number of classes selected from each stratum was approximately proportional to the size of the corresponding student population.

The inclusion criteria were as follows: (1) being a full-time first- or second-year undergraduate student enrolled in a sports-related major at the university; (2) providing informed consent and participating voluntarily; and (3) being able to complete the questionnaire independently. The exclusion criteria were as follows: (1) incomplete questionnaires; (2) an unusually short completion time; (3) patterned or invariant responses; and (4) missing key information or logical inconsistencies. A total of 377 questionnaires were distributed, of which 347 were returned. After 22 invalid questionnaires were excluded, 325 valid responses were retained, yielding an effective response rate of 93.66%. The demographic and sport-related characteristics of the participants are presented in Table 1.

**Table 1:** Demographic and sport-related characteristics of participants (*N* = 325).

Characteristic	Category	*n*	%
Sex	Male	191	58.8
	Female	134	41.2
Grade	First year	178	54.8
	Second year	147	45.2
Age (years)	17–18	102	31.4
	19	128	39.4
	20	78	24
	≥21	17	5.2
Sport specialization	Basketball	62	19.1
	Volleyball	41	12.6
	Football	48	14.8
	Track and field	55	16.9
	Badminton	35	10.8
	Table tennis	31	9.5
	Martial arts	29	8.9
	Other sports	24	7.4
Years of sports training	≤3 years	48	14.8
	4–6 years	96	29.5
	7–9 years	111	34.2
	≥10 years	70	21.5

### Instruments

#### Demographic and sport-related characteristics

Demographic and sport-related information was collected using five self-report items: sex, grade, age, sport specialization, and years of sports training. The distribution of these characteristics is presented in Table 1.

#### Family capital

Family capital was conceptualized as a multidimensional construct comprising economic, cultural, and social capital. Drawing on Bourdieu’s theory of capital and the operational framework of Su et al. (2025), and considering the characteristics of undergraduate sports majors, the family capital measure consisted of nine items across three dimensions.

Economic capital comprised two items: monthly family income and the family’s capacity to afford educational expenses. Monthly family income was coded into five ascending categories: 1 = <5,000 RMB, 2 = 5,000–9,999 RMB, 3 = 10,000–14,999 RMB, 4 = 15,000–19,999 RMB, and 5 = ≥20,000 RMB. The family’s capacity to afford educational expenses was assessed on a five-point scale: 1 = completely unable to afford, 2 = difficult to afford, 3 = basically able to afford, 4 = relatively easy to afford, and 5 = fully able to afford. The two item scores were summed to obtain the economic capital score, with a theoretical range of 2–10. Higher scores indicated greater family economic capital.

Cultural capital comprised four items: paternal educational attainment, maternal educational attainment, family attention to sports events and sports culture, and family sports atmosphere. Paternal and maternal educational attainment were each coded into five categories: 1 = junior high school education or below, 2 = senior high school education, 3 = junior college education, 4 = bachelor’s degree, and 5 = master’s degree or above. Family attention to sports events and sports culture and family sports atmosphere were each rated on a five-point scale ranging from 1 = very low to 5 = very high. The four item scores were summed to obtain the cultural capital score, with a theoretical range of 4–20. Higher scores indicated greater family cultural capital.

Social capital comprised three items: paternal occupational status, maternal occupational status, and the occupational status of close relatives. Based on China’s occupational structure and the International Standard Classification of Occupations (ISCO) (International Labour Office, 2012), each item was coded into five categories: 1 = unskilled workers, agricultural laborers, and other low-skilled occupations; 2 = service workers, general clerical workers, and ordinary operational positions; 3 = skilled workers, technical employees, and front-line management positions; 4 = professionals and middle-level managers; and 5 = senior managers, senior professionals, and other occupations associated with greater social resources. The three item scores were summed to obtain the social capital score, with a theoretical range of 3–15. Higher scores indicated greater family social capital.

The nine item scores were summed to obtain the overall family capital score, yielding a theoretical range of 9–45, with higher scores indicating greater family capital. In the present sample, the observed ranges were 2–10 for economic capital, 4–18 for cultural capital, and 3–15 for social capital. In this study, Cronbach’s α for the overall family capital measure was 0.845. Cronbach’s α values for the economic, cultural, and social capital dimensions were 0.880, 0.866, and 0.832, respectively. The KMO value was 0.816, and Bartlett’s test of sphericity was significant (*p* < 0.001).

#### Academic self-efficacy

This study used the Academic Self-Efficacy Scale developed by Liang (2004) to assess academic self-efficacy among undergraduate sports majors. The scale consists of 22 items across two dimensions: academic ability self-efficacy and academic behavior self-efficacy, with 11 items in each dimension. Academic ability self-efficacy reflects individuals’ judgments of their capacity to complete learning tasks and meet academic requirements. Academic behavior self-efficacy reflects individuals’ confidence in initiating, sustaining, and regulating learning behaviors. All items were rated on a five-point Likert scale ranging from 1 = completely disagree to 5 = completely agree. Higher scores indicated greater academic self-efficacy. In this study, Cronbach’s α for the overall scale was 0.873. Cronbach’s α values for the academic ability and academic behavior self-efficacy dimensions were 0.757 and 0.753, respectively. The KMO value was 0.894, and Bartlett’s test of sphericity was significant (*p* < 0.001).

#### Academic burnout

This study used the College Student Academic Burnout Scale, translated and revised by Lian et al. (2005), to assess academic burnout among undergraduate sports majors. Based on Maslach’s theory of burnout, the scale consists of 20 items across three dimensions: low mood, inappropriate behavior, and low academic achievement (reflecting reduced personal accomplishment rather than objective academic performance). Conceptually, this dimension differs from academic self-efficacy, which concerns students’ beliefs about their capability to perform academic tasks and regulate learning behaviors. Items 1, 3, 6, 8, 11, 13, 15, and 18 were reverse-scored. All items were rated on a five-point Likert scale, with higher total scores indicating greater academic burnout. Following previous applications of the scale (Zhu et al., 2023), participants were classified as having no academic burnout (≤ 40), an uncertain burnout status (41–59), or academic burnout (≥ 60). These categories were used for descriptive purposes only. In this study, Cronbach’s α for the overall scale was 0.920. Cronbach’s α values for the low mood, inappropriate behavior, and low academic achievement dimensions were 0.787, 0.735, and 0.739, respectively. The KMO value was 0.855, and Bartlett’s test of sphericity was significant (*p* < 0.001).

### Procedure

This study was approved by the Ethics Committee of K University (approval number: KM2025125J) and conducted in accordance with the Declaration of Helsinki. Data were collected in March 2026. After students were stratified by grade and sport specialization and eligible classes were randomly selected using the computer-generated procedure described above, course instructors in the selected classes distributed the Wenjuanxing questionnaire link to their class groups. Before completing the questionnaire, all participants read an electronic informed consent statement. Participants proceeded to the formal questionnaire only after being informed of the study purpose, anonymity, and the voluntary nature of participation.

The questionnaire was presented in the following order: informed consent, demographic information, family capital measures, academic self-efficacy scale, and academic burnout scale. All items were mandatory, and completion took approximately 20–30 min. The survey was conducted anonymously. Participants received no material compensation, and each IP address was permitted to submit only one response.

After data collection, responses were screened according to prespecified data quality criteria. Responses were excluded if they were incomplete, had an unusually short completion time (< 300 s), showed patterned or invariant responses, or contained logical inconsistencies or missing key information. All data were used solely for academic research purposes. Participants could withdraw at any time during the survey without adverse consequences.

### Data analysis

Data were analyzed using IBM SPSS Statistics 26.0. First, invalid responses were excluded, and reverse-scored items were recoded. Descriptive statistics, Cronbach’s α coefficients, KMO values, and Bartlett’s tests of sphericity were used to examine sample characteristics and the reliability and validity of the measures. The sample was then divided into low, medium, and high family capital groups based on tertiles of the total family capital score. Pearson correlation analysis was used to examine associations among the main variables, and one-way analysis of variance was used to test group differences. When significant group differences were identified, Tukey’s HSD test was used for *post hoc* comparisons when the homogeneity of variance assumption was satisfied, whereas the Games–Howell test was used when this assumption was violated. Welch’s robust test was additionally reported when homogeneity of variance was not satisfied. Finally, Model 4 of the PROCESS macro was used to test the mediating role of academic self-efficacy, with 5,000 bootstrap resamples used to estimate 95% confidence intervals. The mediating effect was considered significant when the 95% confidence interval did not include zero. All tests were two-tailed, and statistical significance was set at *p* < 0.05.

## Results

### Descriptive statistics

Descriptive statistics showed that 325 valid responses were included in the analysis. The mean family capital score was 23.44 (SD = 6.01), the mean academic self-efficacy score was 61.92 (SD = 11.36), and the mean academic burnout score was 57.19 (SD = 12.23). Detailed descriptive statistics for all variables and dimensions are presented in Table 2.

**Table 2:** Descriptive statistics of the main variables.

Variable	*N*	Minimum	Maximum	Mean	Standard deviation
Economic capital	325	2	10	4.82	2.01
Cultural capital	325	4	18	11.56	3.12
Social capital	325	3	15	7.06	2.73
Family capital	325	10	40	23.44	6.01
Academic ability self-efficacy	325	15	47	30.81	5.86
Academic behavior self-efficacy	325	15	47	31.11	5.86
Academic self-efficacy	325	30	88	61.92	11.36
Low mood	325	11	35	22.86	4.98
Inappropriate behavior	325	9	26	17.17	3.82
Low academic achievement	325	7	28	17.16	3.75
Academic burnout	325	28	88	57.19	12.23

This study used tertile-based grouping. Using the 33.33rd and 66.67th percentiles of the total family capital score as cut-off points (20 and 26, respectively), participants were divided into low (*n* = 109), moderate (*n* = 116), and high (*n* = 100) family capital groups. This quantile-based approach is commonly used to divide continuous variables into relatively balanced comparison groups according to the sample distribution (Altman and Bland, 1994). Based on the scoring criteria of the Academic Burnout Scale, 29 participants were classified as having no academic burnout, 154 as having an uncertain burnout status, and 142 as having academic burnout. The distribution of participants across burnout categories is shown in Figure 1.

**Figure 1:**
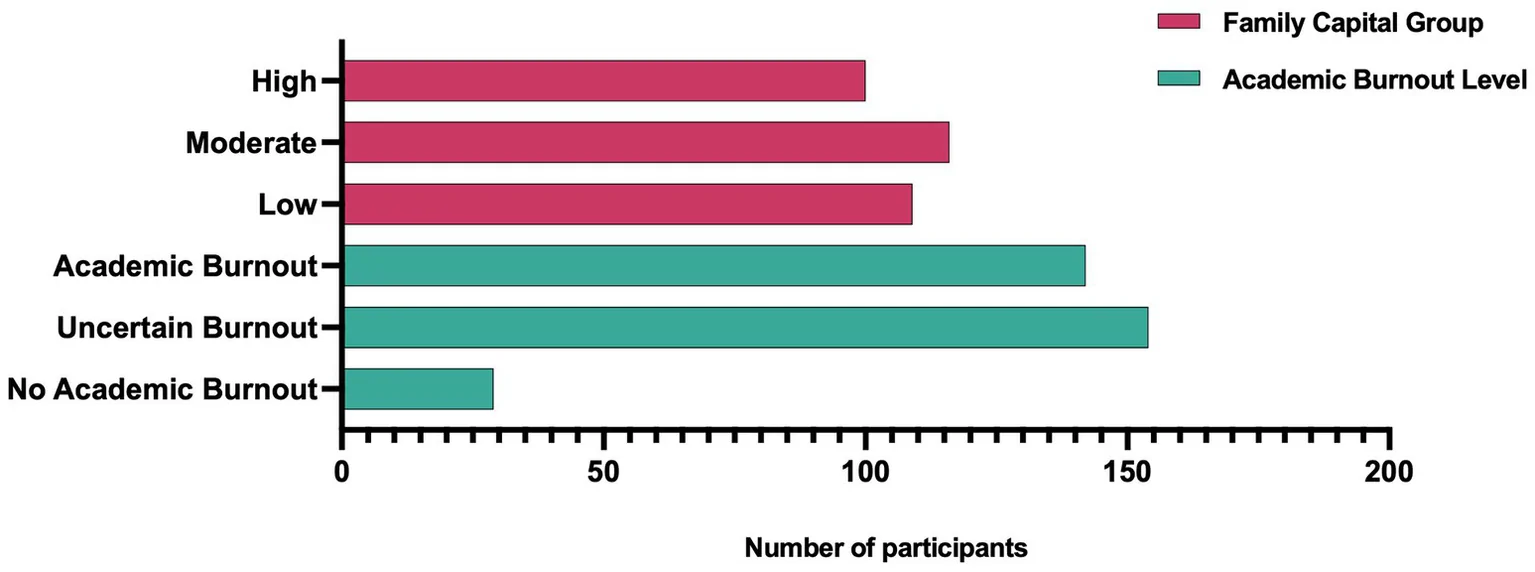
Distribution of participants by family capital group and academic burnout level.

### Correlation analysis

Pearson correlation analysis was conducted to examine associations among family capital, academic self-efficacy, and academic burnout. Family capital was positively correlated with academic self-efficacy (r = 0.554, *p* < 0.001) and negatively correlated with academic burnout (r = −0.575, *p* < 0.001). Academic self-efficacy was negatively correlated with academic burnout (r = −0.637, *p* < 0.001).

Regarding the dimensions of family capital, cultural capital and social capital were positively correlated with academic self-efficacy, r = 0.530 and r = 0.517, respectively, both *p* < 0.001. Cultural capital and social capital were also negatively correlated with academic burnout, r = −0.587 and r = −0.590, respectively, both *p* < 0.001. Economic capital was weakly but positively correlated with academic self-efficacy (r = 0.129, *p* = 0.020), but was not significantly correlated with academic burnout (r = −0.002, *p* = 0.967). The full correlation matrix is presented in Table 3.

**Table 3:** Correlation matrix of the main variables and their dimensions.

Variable	Economic capital	Cultural capital	Social capital	Family capital	Academic ability self-efficacy	Academic behavior self-efficacy	Academic self-efficacy	Low mood	Inappropriate behavior	Low academic achievement	Academic burnout
Economic capital	1										
Cultural capital	0.086	1									
Social capital	0.085	0.751***	1								
Family capital	0.417***	0.891***	0.874***	1							
Academic ability self-efficacy	0.133*	0.522***	0.488***	0.538***	1						
Academic behavior self-efficacy	0.118*	0.506***	0.514***	0.536***	0.877***	1					
Academic self-efficacy	0.129*	0.530***	0.517***	0.554***	0.969***	0.969***	1				
Low mood	−0.017	−0.589***	−0.584***	−0.578***	−0.614***	−0.617***	−0.635***	1			
Inappropriate behavior	0	−0.563***	−0.573***	−0.554***	−0.571***	−0.570***	−0.589***	0.936***	1		
Low academic achievement	0.015	−0.557***	−0.563***	−0.541***	−0.613***	−0.611***	−0.632***	0.926***	0.895***	1	
Academic burnout	−0.002	−0.587***	−0.590***	−0.575***	−0.617***	−0.617***	−0.637***	0.984***	0.969***	0.964***	1

*p < 0.05, ***p < 0.001.

### Between-group difference analysis

To compare academic self-efficacy and academic burnout across family capital groups, normality was first assessed for the low, moderate, and high family capital groups. The Shapiro–Wilk test indicated that academic self-efficacy did not significantly deviate from normality across the three groups, with *p* = 0.923, 0.430, and 0.519, respectively. For academic burnout, the moderate and high family capital groups did not significantly deviate from normality (*p* = 0.054 and 0.182, respectively), whereas the low family capital group showed a slight deviation from normality (*p* = 0.035). Given the relatively large group sizes, one-way analysis of variance was conducted, with Welch’s robust test additionally reported where the homogeneity of variance assumption was not met.

Descriptive statistics showed that mean academic self-efficacy scores were 54.47, 62.90, and 68.91 in the low, moderate, and high family capital groups, respectively. Mean academic burnout scores were 65.80, 56.48, and 48.63, respectively. Means, standard deviations, and between-group comparison results for the dimensions of academic self-efficacy and academic burnout are presented in Table 4.

**Table 4:** Differences in academic self-efficacy and academic burnout among students with different levels of family capital.

Variable	Low family capital group (M ± SD)	Moderate family capital group (M ± SD)	High family capital group (M ± SD)	F	Welch	*p*	η^2^
Academic ability self-efficacy	27.02 ± 4.451	31.35 ± 5.612	34.30 ± 5.078	54.586	62.422	< 0.001	0.253
Academic behavior self-efficacy	27.45 ± 4.742	31.54 ± 5.519	34.61 ± 5.019	51.731	56.773	< 0.001	0.243
Academic self-efficacy	54.47 ± 8.809	62.90 ± 10.704	68.91 ± 9.632	57.869	65.074	< 0.001	0.264
Low mood	26.40 ± 3.356	22.52 ± 5.122	19.40 ± 3.533	75.936	108.055	< 0.001	0.32
Inappropriate behavior	19.72 ± 2.652	17.00 ± 4.082	14.58 ± 2.610	66.589	99.591	< 0.001	0.293
Low academic achievement	19.67 ± 2.539	16.97 ± 3.876	14.65 ± 2.869	65.666	89.915	< 0.001	0.29
Academic burnout	65.80 ± 8.056	56.48 ± 12.738	48.63 ± 8.584	75.516	111.042	< 0.001	0.319

Low family capital group, *n* = 109; moderate family capital group, *n* = 116; high family capital group, *n* = 100. M ± SD represents mean ± standard deviation. Welch’s robust test was additionally reported because the homogeneity of variance assumption was not met for some variables. For post hoc comparisons, Tukey’s HSD test was used when homogeneity of variance was satisfied, whereas the Games–Howell test was used when this assumption was violated. Post hoc comparisons showed significant differences among the three groups for all indicators.

The analysis of variance showed significant differences in academic self-efficacy and academic burnout across family capital groups. Specifically, academic self-efficacy differed significantly across groups, *F* (2, 322) = 57.869, *p* < 0.001, η^2^ = 0.264. Academic burnout also differed significantly across groups, *F* (2, 322) = 75.516, *p* < 0.001, η^2^ = 0.319. Welch’s robust test further confirmed significant group differences for academic self-efficacy (Welch = 65.074, *p* < 0.001) and academic burnout (Welch = 111.042, *p* < 0.001).

Tukey’s HSD *post hoc* comparisons showed that, for academic self-efficacy, the high family capital group scored significantly higher than the moderate family capital group, mean difference = 6.013, *p* < 0.001, 95% CI [2.87, 9.15]. The high family capital group also scored significantly higher than the low family capital group, mean difference = 14.442, *p* < 0.001, 95% CI [11.26, 17.63]. The moderate family capital group scored significantly higher than the low family capital group, mean difference = 8.429, *p* < 0.001, 95% CI [5.36, 11.50].

Games–Howell *post hoc* comparisons showed that, for academic burnout, the low family capital group scored significantly higher than the moderate family capital group, mean difference = 9.315, *p* < 0.001, 95% CI [5.98, 12.65]. The low family capital group also scored significantly higher than the high family capital group, mean difference = 17.168, *p* < 0.001, 95% CI [14.44, 19.89]. The moderate family capital group scored significantly higher than the high family capital group, mean difference = 7.853, *p* < 0.001, 95% CI [4.40, 11.30].

### Regression and mediation effects

To further examine the mechanism linking family capital to academic burnout, this study specified academic self-efficacy as the mediating variable and tested the mediation model using Model 4 of the SPSS PROCESS macro. Bootstrapping with 5,000 resamples was used to estimate 95% confidence intervals (see Figure 2).

**Figure 2:**
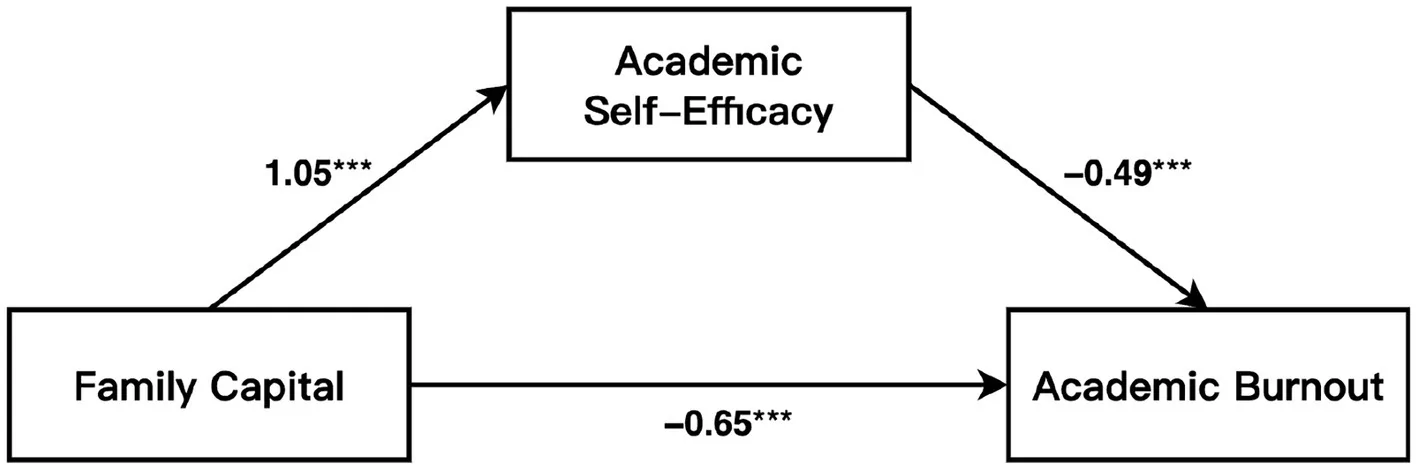
Mediation model of academic self-efficacy between family capital and academic burnout. ***p < 0.001.

Regression analysis showed that family capital had a significant total effect on academic burnout (B = −1.17, t = −12.624, *p* < 0.001, 95% CI [−1.35, −0.99]). Family capital positively predicted academic self-efficacy (B = 1.05, t = 11.972, *p* < 0.001), and academic self-efficacy negatively predicted academic burnout (B = −0.49, t = −9.489, *p* < 0.001). After academic self-efficacy was included in the model, the direct effect of family capital on academic burnout remained significant (B = −0.65, t = −6.606, *p* < 0.001, 95% CI [−0.84, −0.46]), indicating partial mediation.

Bootstrap mediation analysis showed that the indirect effect through academic self-efficacy was −0.52. The direct and indirect effects accounted for 55.67 and 44.33% of the total effect, respectively. The 95% bootstrap confidence interval for the indirect effect did not include zero (95% CI [−0.66, −0.40]), indicating that the mediation effect was statistically significant (see Table 5).

**Table 5:** Mediating effect of family capital on academic burnout through academic self-efficacy.

Effect	Effect value (B)	SE	LLCI	ULCI	Effect size
Total effect	−1.17	0.09	−1.35	−0.99	—
Direct effect	−0.65	0.1	−0.84	−0.46	55.67%
Indirect effect	−0.52	0.07	−0.66	−0.4	44.33%

Bootstrap resampling was performed 5,000 times. 95% CI indicates the 95% confidence interval.

## Discussion

This study found that family capital was significantly associated with lower academic burnout among undergraduate sports majors. Although participants in the present study were enrolled in sports-related degree programs, research on student-athletes provides a relevant comparison for understanding the academic and sport-related demands encountered in such educational contexts. Previous research has shown that student-athletes are chronically exposed to the dual demands of academic study and athletic training, with training load, academic pressure, competitive performance, and physical fatigue jointly increasing burnout risk (Gould and Whitley, 2009). Xu et al. (2025) further found that athletic burnout among university student-athletes predicted academic burnout, suggesting that exhaustion in the training domain may spill over into the academic domain. Extending this line of research, the present study found that higher family capital was associated with lower academic burnout among undergraduate sports majors. This association suggests that family resources may be relevant to students’ academic adaptation, although the present study did not directly assess training–academic pressure or test whether family capital buffers its effects. In a longitudinal study of student-athletes, Saarinen et al. (2025) found that parental emotional support and success expectations shaped the developmental trajectories of athletic burnout and school burnout. Dorsch et al. (2016) also found that positive parental involvement in student-athletes’ academic and athletic lives was associated with stronger academic self-efficacy and developmental adaptation. Together, these findings extend previous burnout research by highlighting family resources as an additional contextual factor associated with academic adaptation among undergraduate sports majors.

It is notable that economic capital was not significantly associated with academic burnout. This finding is not fully consistent with studies suggesting that insufficient resources increase students’ risk of burnout. One possible explanation is that material resources alone may be less directly related to students’ academic burnout than other forms of family resources. Pennington and Castor (2022) noted that burnout among student-athletes is often associated with competitive pressure, highly professionalized training environments, and excessive time demands. Parental pressure, coaching style, academic burden, and time-management difficulties have also been identified as important sources of burnout among student-athletes (Dubuc-Charbonneau and Durand-Bush, 2018; Thompson et al., 2024). The present findings therefore complement international research focused mainly on training, time pressure, coaching, and self-regulation by highlighting the relevance of family resources to burnout among undergraduate sports majors. This dimension-specific result further suggests that economic capital did not display the same pattern as cultural and social capital. The mechanisms underlying this difference require further investigation.

Compared with economic capital, family cultural capital and social capital were more strongly associated with academic burnout. Cultural capital may provide students with educational expectations, interpretive frameworks, and familiarity with academic and professional development, whereas social capital may facilitate access to information, guidance, social networks, and career-related resources. Families with higher social capital may also provide informational resources related to further education, employment, competition, and career development, thereby reducing students’ sense of helplessness when facing future uncertainty. A sense of belonging has been associated with burnout and graduation expectations among student-athletes, highlighting the importance of supportive relationships for student-athlete adaptation (Fearon et al., 2011). These findings suggest that academic burnout was more strongly associated with cultural and social resources than with material resources alone, highlighting the importance of distinguishing among different forms of family capital rather than treating family capital as a homogeneous construct.

The between-group analysis further supports this interpretation. Students in the low family capital group reported higher levels of academic burnout than those in the moderate and high family capital groups, and these differences were reflected across all three dimensions: low mood, inappropriate behavior, and perceived low academic achievement. This finding extends the correlational results by showing that differences in family capital were reflected not only in overall burnout but also in its emotional, behavioral, and self-evaluative components. Moreover, the graded differences across the low, moderate, and high family capital groups suggest that variation in family resources was systematically associated with students’ academic adjustment rather than being relevant only to an extreme-risk subgroup. Saarinen et al. (2025) found that lower success expectations were associated with higher levels of athletic and school burnout. Together, these findings indicate that differences in family resources may be reflected across multiple aspects of students’ burnout experiences.

This study also found that academic self-efficacy partially mediated the association between family capital and academic burnout. In other words, family capital may be linked to lower burnout by strengthening students’ beliefs in their learning ability and problem-solving capacity. This finding goes beyond the direct association between family capital and burnout by identifying academic self-efficacy as one psychological pathway through which family resources may be related to students’ academic adaptation. Greater family resources may provide access to guidance, information, and problem-solving support that strengthen students’ confidence in managing academic demands. This finding is consistent with Dorsch et al. (2016), who found that parental involvement was associated with stronger academic self-efficacy among student-athletes. In addition, Öztürk Çelik (2025) found that self-efficacy was closely associated with burnout and mental health among student-athletes. Stronger academic self-efficacy may in turn support persistence and more adaptive responses to academic difficulties, thereby reducing vulnerability to burnout.

However, academic self-efficacy only partially mediated this association, suggesting that family capital may be linked to academic burnout through multiple pathways. Beyond academic self-efficacy, resource-related factors such as access to career information, as well as broader family and educational processes such as parental emotional support, professional identity formation, coach–student relationships, and peer support, may also be relevant. These factors were not directly assessed in the present study and should be examined in future research. The partial mediation therefore indicates that academic self-efficacy explains only part of the association between family capital and academic burnout and that a broader framework incorporating family resources, psychological beliefs, and contextual factors may be needed to more fully understand burnout among undergraduate sports majors.

This study contributes to the literature by extending research on academic burnout among undergraduate sports majors from a predominantly training- and school-related perspective to a family resource perspective. By distinguishing the economic, cultural, and social dimensions of family capital and identifying academic self-efficacy as a partial mediator, the study further shows that different forms of family resources may be associated with academic adaptation through both resource-based and psychological pathways. In practice, university physical education departments should pay particular attention to students with limited cultural and social resources and lower academic self-efficacy. Academic guidance, self-efficacy–enhancing learning support, access to educational and career information, and opportunities to strengthen students’ social and informational resources may help support their academic adaptation (Wang et al., 2025).

This study has several limitations. First, the cross-sectional survey design can identify associations among variables but cannot establish causal relationships. Future research should use longitudinal designs to examine the dynamic relationships among family capital, academic self-efficacy, and academic burnout. Second, the sample was drawn from a single sports university, which limits the generalizability of the findings to students from other regions, institutional levels, and sports specializations. Future studies should include larger and more diverse samples across multiple institutions and regions. Third, family capital was assessed based on students’ self-reports, and students may not have complete or fully accurate knowledge of household income or the occupational circumstances of their parents and close relatives. In addition, family capital, academic self-efficacy, and academic burnout were all reported by the same respondent, which may introduce shared-source bias. Future research could incorporate parent-reported family information, objective or administrative indicators, and multi-informant data to improve measurement accuracy. Finally, this study did not fully incorporate sport-specific contextual variables, such as training load, injury history, competition level, coach support, peer relationships, and professional identity. Future research could combine interviews, training performance data, and course grades to clarify how family capital is related to learning confidence and psychological adaptability among sports majors.

## Conclusion

This study addressed its objectives by examining the associations among family capital, academic self-efficacy, and academic burnout among undergraduate sports majors, comparing the associations across different dimensions of family capital, and testing the mediating role of academic self-efficacy. Overall family capital was negatively associated with academic burnout and positively associated with academic self-efficacy, while academic self-efficacy was negatively associated with academic burnout. Accordingly, H1, H2, and H3 were supported. Academic self-efficacy also partially mediated the association between overall family capital and academic burnout, providing support for H4. The dimension-specific analyses further showed that cultural and social capital were significantly associated with lower academic burnout and higher academic self-efficacy, whereas economic capital was only weakly associated with academic self-efficacy and was not significantly associated with academic burnout. These findings indicate that different forms of family capital are not equally associated with academic adaptation and directly address the study objective of examining differences across family capital dimensions. Taken together, the findings suggest that family capital, particularly cultural and social resources, is associated with academic burnout among undergraduate sports majors, with academic self-efficacy representing a partial mediating pathway. Universities should therefore pay particular attention to students with limited cultural and social resources and lower academic self-efficacy and provide targeted academic guidance, learning support, and access to educational and career-related information.

## Data Availability

The original contributions presented in the study are included in the article/supplementary material, further inquiries can be directed to the corresponding authors.
